# Sleep wake related changes in intracellular chloride regulate plasticity at glutamatergic cortical synapses

**DOI:** 10.1016/j.cub.2025.01.050

**Published:** 2025-02-21

**Authors:** Hannah Alfonsa, Atreyi Chakrabarty, Vladyslav V. Vyazovskiy, Colin J. Akerman

**Affiliations:** 1 Department of Pharmacology, University of Oxford, Mansfield Road, Oxford, OX1 3QT, United Kingdom; 2 Department of Physiology, Anatomy and Genetics, University of Oxford, Sherrington Road, Oxford, OX1 3PT, United Kingdom; 3 Sir Jules Thorn Sleep and Circadian Neuroscience Institute, University of Oxford, South Park Road, Oxford, OX1 3QU, United Kingdom; 4 The Kavli Institute for Nanoscience Discovery, University of Oxford, Sherrington Road, Oxford, OX1 3QU, United Kingdom

## Abstract

Wakefulness and sleep affect the brain’s ability to exhibit plastic changes^1,2^. For instance, the potentiation of cortical excitatory synaptic connections is associated with the active period, when animals are mainly awake^3–7^. It is unclear, however, how changes in neuronal physiology that are associated with sleep-wake history, affect the mechanisms responsible for synaptic plasticity. Recently it has been shown that sleep-wake history alters transmembrane chloride (Cl^-^) gradients in cortical pyramidal neurons via Cl^-^ co-transporter activity, which shifts the reversal potential for GABA_A_ receptors (E_GABAA_) when assessed *in vivo* and *in vitro*^8,9^. Hyperpolarizing E_GABAA_ values are associated with recent sleep, whilst depolarizing E_GABAA_ values are associated with recent waking. Here we demonstrate that sleep-wake history related changes in E_GABAA_ affect membrane potential dynamics and glutamatergic long-term potentiation (LTP) elicited by spiking activity in pyramidal neurons of mouse cortex. Reducing the depolarized shift in E_GABAA_ during the active period reduces the potentiation of cortical excitatory synapses onto layer 5 (L5) pyramidal neurons. Depolarized E_GABAA_ values facilitate LTP induction by promoting residual membrane depolarization during synaptically-evoked spiking. And changes in LTP induction associated with sleep-wake history can be reversed by switching the E_GABAA_-dependent effects, either by using direct current injection to counteract the effects upon residual membrane potential depolarization, or by modulating co-transporters that regulate E_GABAA_. We conclude that E_GABAA_ dynamics provide a functional link between changes in a neuron’s physiology that are associated with sleep-wake history, and the mechanisms responsible for the induction of glutamatergic synaptic plasticity.

## Results and Discussion

### Depolarized E_GABAA_ contributes to excitatory synaptic potentiation during the active period

**Figure 1:**
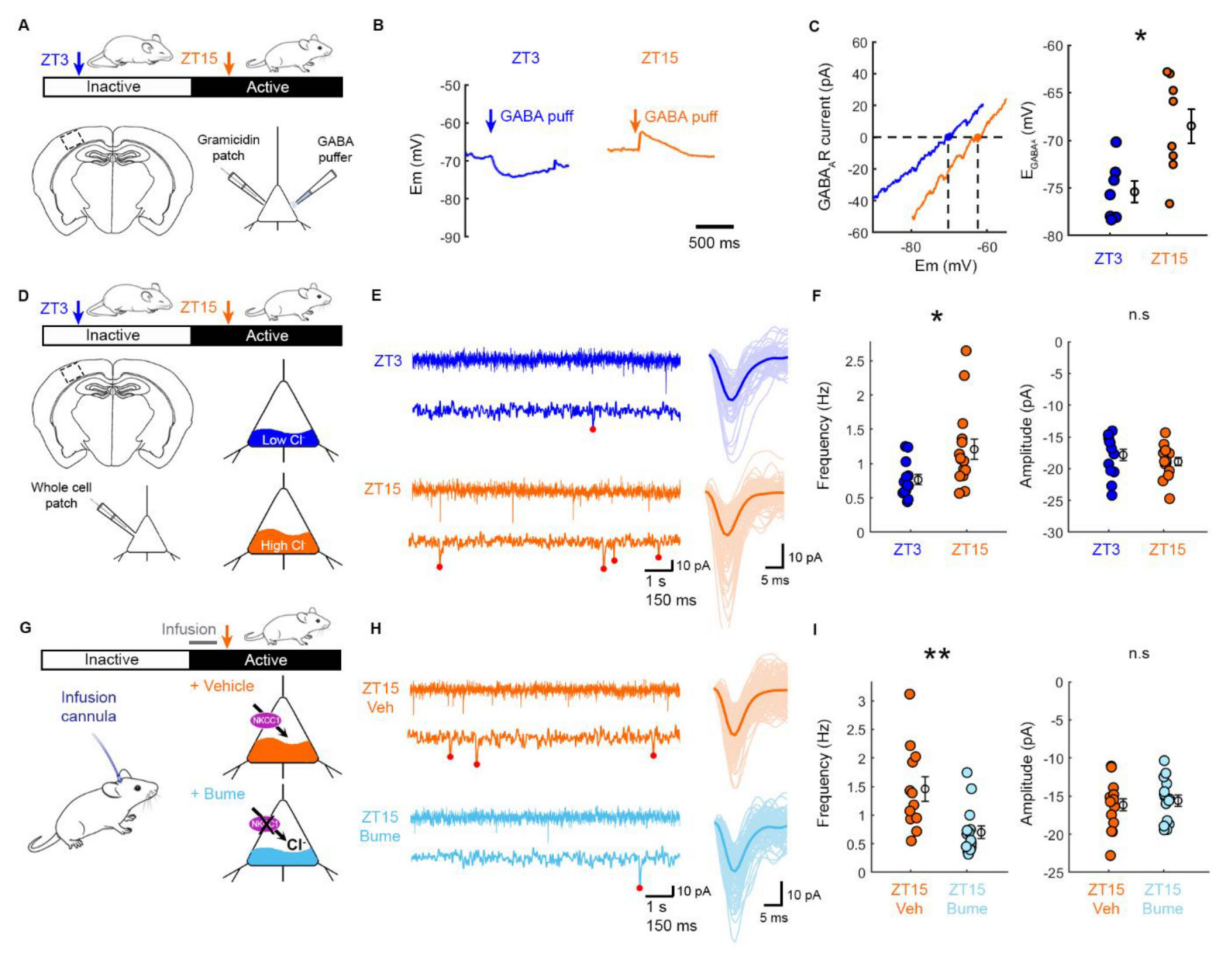
Depolarized E_GABAA_ contributes to an increase in miniature excitatory post-synaptic currents during the active period. (A) Brain slices were prepared from S1 at ZT3 during the inactive period when mice tend to sleep, or at ZT15 during the active period when mice tend to be awake. Gramicidin perforated recordings compared GABA_A_R signalling in L5 pyramidal neurons. See Figure S1. (B) Current clamp recordings show the effect of GABA_A_R activation. (C) GABA_A_R IV curves from ramp protocols in voltage clamp mode. E_GABAA_, the membrane potential at which the GABA_A_R current equals zero, was more depolarized at ZT15 compared to ZT3 (7 and 8 neurons, 3 and 5 animals; *p=0.014, Mann-Whitney; d=1.62). (D) Whole-cell recordings monitored mEPSCs. (E) Recordings (left) from ZT3 and ZT15 showing detected mEPSCs (right). (F) mEPSC frequency was higher at ZT15 than ZT3 (13 and 19 neurons, 3 and 4 animals; *p=0.015, t-test; t=2.597; df=27; d=0.827). No difference in mEPSC amplitude (13 and 19 neurons, 3 and 4 animals; p=0.9921, t-test; t=0.01; df=27). (G) Vehicle or bumetanide was infused into S1 at the beginning of light offset, when animals are typically awake. Arrow indicates when slices were prepared. (H) Recordings from ZT15-Veh and ZT15-Bume conditions. (I) mEPSC frequency was reduced by bumetanide (12 and 15 neurons, 3 and 4 animals; **p=0.003, t-test; t=3.365; df=25; d=1.303). No difference in mEPSC amplitude (12 and 15 neurons, 3 and 4 animals; p=0.835, t-test; t=0.21; df=25).

Mice tend to spend the majority of time asleep during the light (inactive) period and the majority of time awake during the dark (active) period, especially in the first few hours after dark onset^10^. In line with recent work^8,9^, we assessed E_GABAA_ at two time points within the 24 h cycle that correspond to either the inactive period (Zeitgeber time 3; ZT3), or the active period (ZT15) (Figure 1A). Acute slices of primary somatosensory cortex (S1) were prepared and L5 pyramidal neurons were targeted for gramicidin perforated patch clamp recordings, which preserve transmembrane Cl^-^ gradients (Figure 1A)^11,12^. Consistent with evidence from multiple cortical areas^8^, GABA_A_Rs typically elicited hyperpolarizing responses at ZT3, but depolarizing responses at ZT15 (Figure 1B), and voltage ramp protocols confirmed that E_GABAA_ was more depolarized at ZT15 (Figure 1C). No difference was observed in the resting membrane potential and, as a result, the GABA_A_R driving force was also more depolarized at ZT15 (Figure S1).

In addition to more depolarized E_GABAA_ values, the active period is associated with a net potentiation of cortical excitatory synaptic connections^3–7^. Consistent with this, when we measured miniature excitatory post-synaptic currents (mEPSCs; Figure 1D), we observed a higher mEPSC frequency at ZT15 compared to ZT3 (Figure 1E-F)^5,6^. To assess whether the depolarized E_GABAA_ at ZT15 contributes to the potentiation of excitatory synaptic connections, we blocked the Cl^-^ co-transporter, NKCC1, which is upregulated during the active period and contributes to the more depolarized E_GABAA_ in pyramidal neurons^8^. The NKCC1 blocker, bumetanide, was infused *in vivo* into S1 from the start of the active period and slices were prepared at ZT15 (Figure 1G). mEPSC frequency was found to be reduced following bumetanide treatment compared to vehicle control (Figure 1H-I). These data suggest that depolarized E_GABAA_ contributes to an increase in mEPSC frequency observed at ZT15, and implicate E_GABAA_ in the net potentiation of excitatory synaptic connections associated with the active period^3–7^.

### E_GABAA_ effects upon membrane potential dynamics generate differences in LTP induction

**Figure 2:**
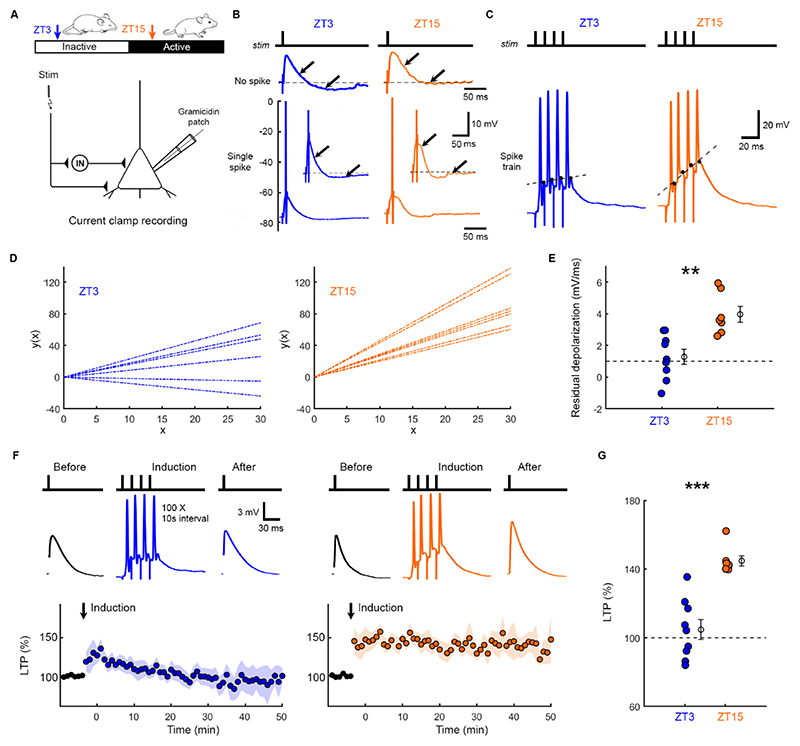
Depolarized E_GABAA_ during active period is associated with residual membrane depolarization during synaptically-evoked spiking and glutamatergic synaptic LTP. (A) Gramicidin recordings monitored membrane potential responses in L5 pyramidal neurons following activation of an excitatory-inhibitory circuit via a stimulating electrode in L2/3. See Figure S2. (B) Recordings when stimulus intensity was below (top) or above (bottom) the threshold to elicit a post-synaptic spike. Arrows at corresponding timepoints indicate how disynaptic IPSPs differentially affect membrane potential dynamics. (C) Responses to a high frequency pre-synaptic stimulus train (four pulses at 100 Hz) revealed greater residual depolarization between action potentials in ZT15 neurons, defined as the slope of the linear fit to the post-spike depolarization. (D) Population data showing linear fits to residual depolarization. (E) Residual depolarization was greater at ZT15 (9 and 7 neurons, 7 and 5 animals; **p=0.002, t-test; t=3.91; df=14; d=1.97). (F) LTP (EPSP peak amplitude, normalized to baseline) was monitored before and after induction, which involved eliciting trains of synaptically-evoked postsynaptic spikes (four pulses at 100 Hz, repeated 100 times at 0.1 Hz). (G) LTP was larger at ZT15 compared to ZT3 (9 and 7 neurons, 7 and 5 animals; when measured 5-15 min post-induction ***p=0.0002, Mann-Whitney; d=2.86 or 0-20 min post-induction **p=0.0052, Mann-Whitney; d=1.82). See Figure S3.

To investigate how changes in E_GABAA_ might affect glutamatergic synaptic potentiation, we examined how L5 pyramidal neurons respond to a canonical cortical circuit motif comprising monosynaptic excitation and disynaptic inhibition (Figure 2A and Figure S2)^13–15^. Consistent with their more hyperpolarized E_GABAA_, gramicidin recordings at ZT3 revealed more hyperpolarized potentials during the disynaptic inhibitory window following stimulation. This was apparent when the post-synaptic neuron did not reach action potential threshold, and when the stimulus intensity was increased to a suprathreshold level (Figure 2B). In contrast, and consistent with their more depolarized E_GABAA_, ZT15 neurons tended to remain more depolarized and showed less hyperpolarization during the disynaptic inhibitory window (Figure 2B). These differences between ZT3 and ZT15 were also evident when the suprathreshold stimulus was used to evoke trains of postsynaptic spiking activity (Figure 2C; 100 Hz, 4 pre-synaptic stimuli), such as those shown to elicit LTP at excitatory synapses onto L5 pyramidal neurons^16^. Consistent with their more depolarized E_GABAA_ and its effects during the disynaptic inhibitory window, neurons at ZT15 exhibited greater residual depolarization between synaptically-evoked action potentials^16,17^, which increased over the train (Figure 2C). This residual depolarization could be quantified as the slope of the linear fit to the post-spike depolarization (Figure 2C and 2D), and was greater at ZT15 than at ZT3 (Figure 2E).

Postsynaptic membrane potential, including residual depolarization between postsynaptic spikes, has been identified as a critical determinant of LTP induction at L5 glutamatergic synapses^16–19^. We therefore hypothesised that differences in residual depolarization between ZT3 and ZT15 may be associated with differences in LTP induction. Using gramicidin recordings in L5 pyramidal neurons, monosynaptic EPSP peak amplitude was monitored in response to presynaptic input from L2/3 and compared before and after an LTP induction protocol. Induction involved eliciting synaptically-evoked trains of high frequency postsynaptic spikes (4 pre-synaptic stimuli at 100 Hz, as in Figure 2C), repeated 100 times at an interval of 10 s (Figure 2F). This protocol was found to induce a greater level of LTP in slices prepared at ZT15 than at ZT3 (Figure 2G). 100% of the neurons at ZT15 exhibited potentiation, compared to only 22% of neurons at ZT3, and the difference between ZT15 and ZT3 was evident when the EPSP amplitude or slope was used to quantify LTP (Figure S3A). Control experiments confirmed that the induction protocol elicited equivalent numbers of postsynaptic spikes at ZT15 and ZT3 (9 and 7 neurons, 7 and 5 animals; p=0.965, t-test) and did not affect E_GABAA_ (Figure S3B).

**Figure 3:**
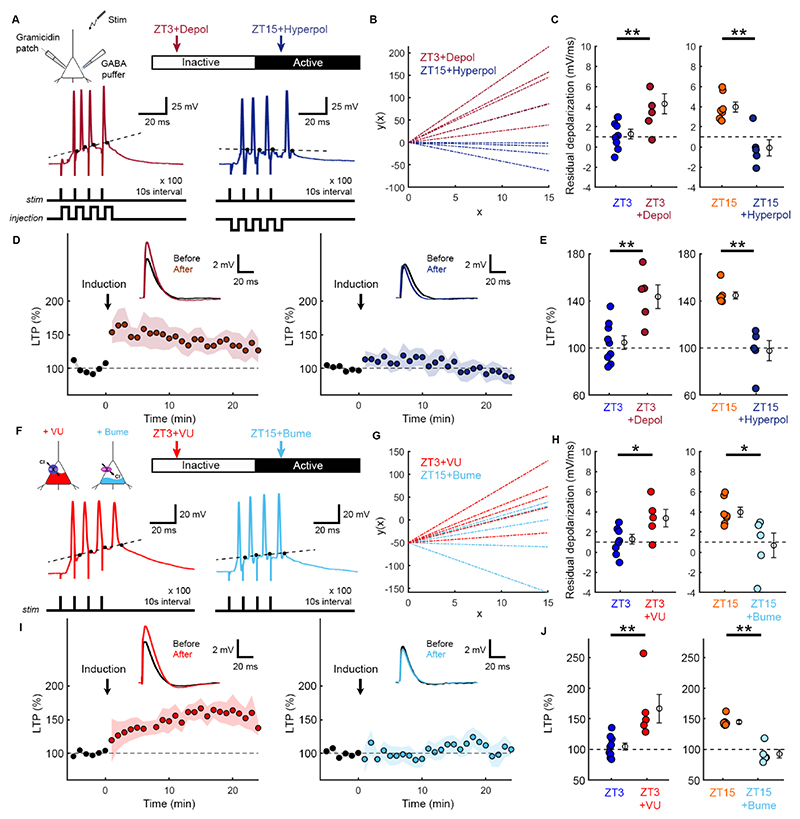
E_GABAA_ effects upon residual membrane depolarization during synaptically-evoked spiking underlies differences in LTP induction between ZT3 and ZT15. (A) Gramicidin recordings were used to monitor synaptically-evoked spike trains, whilst brief somatic current pulses (4 ms) were injected to modulate the membrane potential between spikes. (B) Linear fits to the residual depolarization. (C) Residual depolarization increased in the ZT3+Depolarization condition compared to ZT3 (control from Figure 2E; 5 neurons, 5 animals; **p=0.009, t-test; t=3.109; df=12; d=1.73). Depolarization was reduced in the ZT15+Hyperpolarization condition compared to ZT15 (control from Figure 2E; 5 neurons, 3 animals; **p=0.001, t-test; t=4.5; df=10; d=2.64). (D) Same stimulus train was used to induce LTP (four pulses at 100 Hz, repeated 100 times at 0.1 Hz). (E) LTP was enhanced at ZT3 by increasing the post-spike depolarization during induction (control from Figure 2G; 5 neurons, 5 animals; **p=0.003, t-test; t=3.64; df=12; d=2.03). LTP was attenuated at ZT15 by reducing the post-spike depolarization during induction (control from Figure 2G; 5 neurons, 3 animals; **p=0.003, Mann-Whitney; d=3.48). (F) Gramicidin recordings in KCC2 blocker (VU) at ZT3, or an NKCC1 blocker (Bume) at ZT15. (G) Linear fits to residual depolarization. (H) VU increased residual depolarization at ZT3 (5 neurons, 4 animals; *p=0.017, t-test; t=2.782; df=12; d=1.55). Bumetanide reduced residual depolarization at ZT15 (5 neurons, 4 animals; *p=0.018, t-test; t=2.816; df=10; d=1.65). (I) LTP population data. (J) LTP was enhanced by VU at ZT3 (5 neurons, 4 animals; **p=0.002, Mann-Whitney; d=1.88). LTP was attenuated by bumetanide at ZT15 (5 neurons, 4 animals; **p=0.003, Mann-Whitney; d=4.62).

To examine whether LTP was related to the amount of residual membrane depolarization during induction (Figure 2E), we injected brief depolarizing or hyperpolarizing current pulses between the synaptically-evoked postsynaptic spikes at ZT3 and ZT15, respectively (Figure 3A). Under both of these conditions, we found a striking reversal in the level of residual membrane depolarization and the amount of LTP (Figure 3A-E). Pairing depolarizing current pulses with the synaptically-evoked spiking protocol enhanced the induction of LTP at ZT3 compared to control recordings (Figure 3D-E). In contrast, pairing hyperpolarizing current pulses with the synaptically-evoked spiking protocol reduced the LTP that was elicited at ZT15 (Figure 3D-E).

As a further test of the association between E_GABAA_, residual depolarization and LTP, we used Cl^-^ co-transporter blockers to either produce a depolarizing shift in E_GABAA_ at ZT3 with an antagonist of the Cl^-^ extruder, KCC2 (VU0463271, 10 μm), or a hyperpolarizing shift in E_GABAA_ at ZT15 with an antagonist of the Cl^-^ intruder, NKCC1 (bumetanide, 10 μm)^8,20–24^. Blocking KCC2 to cause a depolarizing shift in E_GABAA_ at ZT3 (ZT3+VU) increased the L5 pyramidal neuron’s residual depolarization during LTP induction protocol (Figure 3F-H). Meanwhile blocking NKCC1 to cause a hyperpolarizing shift in E_GABAA_ at ZT15 (ZT15+Bume) resulted in reduced residual depolarization during the LTP induction protocol (Figure 3F-H). These experiments therefore support the conclusion that Cl^-^ co-transporters and E_GABAA_ contribute to the differences in residual depolarization observed at ZT3 and ZT15. Furthermore, these manipulations also reversed the amount of LTP that was elicited. Blocking KCC2 at ZT3 resulted in greater LTP compared to control recordings at the same time of day (Figure 3I-J). Meanwhile blocking NKCC1 at ZT15 resulted in reduced LTP compared to control recordings at the same time of day (Figure 3I-J).

### Preceding sleep-wake history determines E_GABAA_ and its effects upon LTP induction

**Figure 4:**
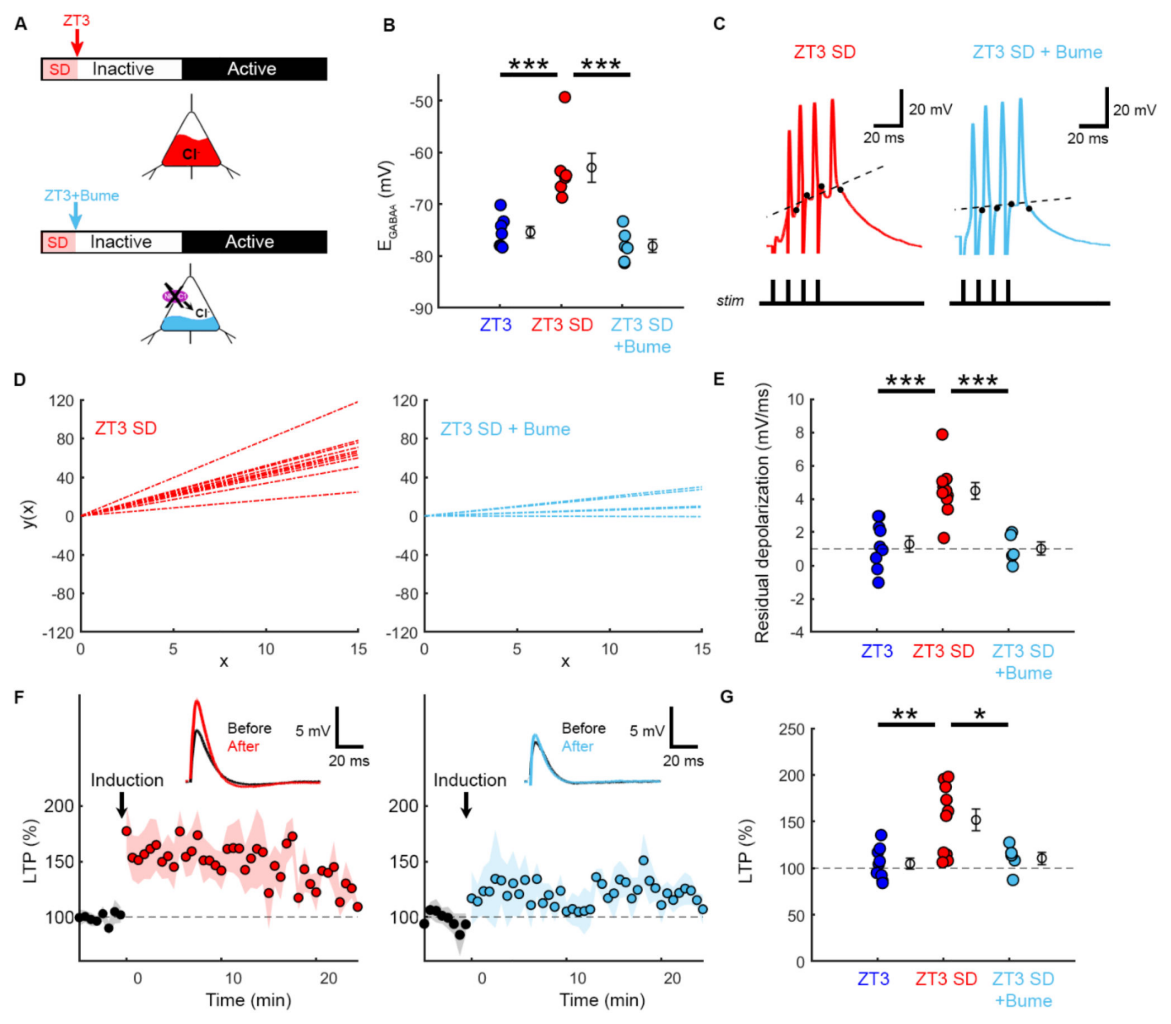
Preceding sleep-wake history determines variations in E_GABAA_ and LTP induction. (A) Animals were exposed to a 3-hour sleep deprivation (SD) protocol at the beginning of the light period and gramicidin recordings were performed under control conditions (ZT3 SD) or under NKCC1 blockade (ZT3 SD+Bume). (B) E_GABAA_ was more depolarized following SD, which was reduced by Bume (control from Figure 1C; 6 neurons, 3 animals; p<0.0001, one-way ANOVA, ***p<0.0001, Tukey-Kramer). (C) Responses to the same stimulus train used to induce LTP (four pulses at 100 Hz). (D) Linear fits to residual depolarization. (E) Residual depolarization was greater in ZT3 SD than ZT3 or ZT3 SD+Bume (control from Figure 2E; 6 neurons, 3 animals; p<0.0001, one-way ANOVA, ***p<0.001, Tukey-Kramer). (F) LTP population data. (G) LTP was higher after SD, which was attenuated by bumetanide (control from Figure 2G; 6 neurons, 3 animals; p<0.0001, one-way ANOVA, **p<0.01 *p<0.05, Tukey-Kramer).

We next investigated whether the differences between ZT3 and ZT15 reflect an animal’s preceding sleep-wake history. We performed a 3-hour sleep deprivation protocol at the beginning of the light period (ZT3 SD) to compare LTP induction at the same time point in the 24-hour cycle, but following different recent sleep-wake histories (Figure 4A). In line with previous work, E_GABAA_ was more depolarized in the sleep-deprived animals^8^ and could be reduced by blocking NKCC1 with bumetanide (Figure 4A-B). At the population level, neurons at ZT3 SD exhibited greater residual depolarization during LTP induction (Figure 4C-E) and greater LTP compared to ZT3 (Figure 4F-G), consistent with a lowering of the threshold for LTP induction in the sleep-deprived animals. Interestingly, a subset of ZT3 SD neurons failed to exhibit LTP (60% of the neurons at ZT3 SD showed potentiation, compared to 100% at ZT15), indicating that LTP induction may be limited by saturation mechanisms that have been observed following extended wakefulness^4,25^. Finally, applying bumetanide at ZT3 SD reduced residual depolarization during the LTP induction protocol and attenuated LTP, consistent with a key role for E_GABAA_ and Cl^-^ regulatory mechanisms in regulating LTP induction (Figure 4C-G). Together, these data support the conclusion that preceding sleep-wake history can account for variations in E_GABAA_, with more depolarized E_GABAA_ being associated with recent wakefulness and enhancing residual membrane depolarization, which facilitates LTP induction.

In summary, our findings extend previous work^8^ by revealing that E_GABAA_ changes associated with sleep-wake history are an important factor regulating LTP induction. Across experimental conditions we observe robust positive correlations between a neuron’s E_GABAA_, the degree of residual membrane depolarization during synaptically-evoked spiking, and the LTP induced by synaptically-evoked spiking (Figure S4). While our data do not rule out the contribution of other factors, we show that modulating E_GABAA_ and its effects upon membrane potential dynamics, either pharmacologically or electrically, is sufficient to regulate LTP induction at different times of day. A defining feature of LTP is that its induction exhibits cooperativity^26^, which has traditionally been considered in terms of excitatory inputs that combine to satisfy induction rules in the postsynaptic neuron, such as achieving sufficient depolarization to relieve the voltage-dependent magnesium block of NMDAR receptors (NMDARs)^27,28^. Our data highlight that cooperativity should be considered in terms of both the glutamatergic and GABAergic synaptic inputs received by a neuron^16,26,28,29^, and E_GABAA_’s effect upon residual depolarization during postsynaptic spiking would be well-placed to regulate the voltage-dependent block of NMDARs. This could be through direct effects on the membrane potential such as observed during development^30–32^, or via indirect effects upon action potential backpropagation^33^, most likely through sodium channel activation mechanisms^16,33^.

Our experiments used an *in vitro* model of synaptically-evoked cortical LTP and it will be interesting to investigate the potential contribution of sleep-wake history E_GABAA_ changes to synaptic plasticity in different brain regions, both *in vitro* and *in vivo*. If they generalize, our observations could hold significance for theories that link sleep and wake to synaptic plasticity and learning processes^1,2^. For example, the synaptic homeostasis hypothesis proposes that encoding information during wakefulness leads to a net increase in synaptic strength, and that sleep is then required to reset synaptic strength^1,34^. The depolarized shifts in E_GABAA_ could represent a cellular mechanism through which excitatory synaptic transmission is increasingly potentiated with wakefulness^5,6,35^. Indeed, one effect of depolarized E_GABAA_ shifts may be to enable LTP induction at times when excitatory synaptic transmission is becoming increasingly saturated^4,25,34,36,37^. Equally, other work has implicated LTP in consolidating learning and memory processes in various brain regions during sleep^2,38–40^. Consistent with this, the effects of depolarized E_GABAA_ upon cortical activity can be observed following the onset of sleep^8^, suggesting that E_GABAA_ is relatively slow to recover following a change in vigilance state, and that depolarized E_GABAA_ could facilitate consolidation processes by lowering the threshold for LTP during early sleep.

### Resource availability

#### Lead contact

Further information and requests for resources and reagents should be directed to and will be fulfilled by the lead contact, Colin Akerman (colin.akerman@pharm.ox.ac.uk).

#### Materials availability

No new materials were generated in this study.

## Methods

### Experimental model and Study Participant Details

Experiments were performed on male C57BL/6 wild-type mice purchased from Charles River and Envigo. Female mice were not used to minimise potential variance due to the effects of the estrous cycle upon sleep and circadian processes^41–43^, although we have no reason to expect sex differences in the relationship between E_GABAA_, residual depolarization, and LTP. Animals were maintained under a 12-h:12-h light-dark (LD) cycle to ensure entrainment. Our previous study used sleep recordings under these conditions to confirm that the animals are well entrained, spending the majority of time asleep during the light (inactive) period and the majority of time awake during the dark (active) period^8,9^. Animals were fed ad libitum. Ambient room temperature was maintained at 22 ± 2 °C and humidity at 50 ± 20%. Animals were aged 4-10 weeks and animal numbers are provided in the figure legends for each experiment. Animals from the same litter were randomly assigned to experimental groups. All experiments were performed in accordance with the United Kingdom Animal Scientific Procedures Act 1986 under personal and project licences granted by the United Kingdom Home Office. Approval was also provided by an Ethical Review Panel at the University of Oxford.

#### Cannula implantation

Cannula implantation was performed at 4-6 weeks of age using stereotactic surgery, aseptic technique, isoflurane anaesthesia (3-5% for induction and 1-2% for maintenance) and constant body temperature monitoring. Analgesia was provided at the beginning of surgery and during recovery (buprenorphine 01.mg/kg and meloxicam 5 mg/kg). The scalp was shaved (Wahl) and cleaned (Hibiscrub). Eye protecting ointment (Viscotears) was applied. A craniotomy was performed over the left hemisphere (AP -1.2 mm, ML -3 mm from Bregma), a 26 gauge single guide cannula (Protech International) was implanted into L5 of left somatosensory cortex (700 nm depth) and a dummy cannula (Protech International) was inserted and secured in to the guide cannula. One self-tapping bone screw (Fine Science Tools) was implanted on the other hemisphere as an anchor. All implants were secured using a non-transparent dental cement (Super-Bond). Animals were singly housed post-surgery and allowed to recover for at least 1 week before experiments.

#### In vivo drug infusion

Stock solutions were made by dissolving bumetanide (Tocris) in DMSO to reach a concentration of 10 mM, aliquoted and stored at -20 °C. Tubing and internal cannulae (Protech International) were flushed with 70% ethanol and sterile saline the day before the experiment. On the day of the experiment, bumetanide were prepared by dissolving stock solutions in sterile saline to reach a final concentration of 55 µM. The vehicle contained the same concentration of DMSO in sterile saline. Each infusion solution was loaded into a heavy wall polyethylene tubing (PE50 - C313CT; Protech International) that allowed the animal to move freely, and was connected to an internal cannula (C315I; Protech International) and captive collar (Protech International). A small air bubble was loaded into the tubing, just before connecting to a 1 µl Hamilton syringe (26 gauge needle). In awake mice, the internal cannula was inserted into the implanted cannula at the beginning of light offset by unscrewing the dummy cannula, gently restraining the animal and covering the eyes to evoke brief freezing behavior. The captive collar was then tightened to secure the internal cannula to the implanted cannula. Infusion was performed using an infuse/withdrawal pump (Harvard apparatus; 700 nl at a speed of 40 nl/min) and monitored by tracking the progression of the air bubble through the tubing. Once the infusion experiment was completed, the internal cannula was disconnected and the dummy cannula replaced. The order of vehicle and drug infusions was counterbalanced and randomized.

#### Sleep deprivation

An established sleep deprivation (SD) protocol was used, which consisted of exposing the animal to novel objects under continuous observation by an experimenter, for a 3-hour period at the beginning of the light period (ZT3 SD). Once an animal stopped exploring an object, a new object was presented. It has been shown that this protocol results in the animal being awake for >98% of the SD period^8^.

#### Acute brain slices

Acute cortical brain slices were prepared for electrophysiological recordings at ZT3 or ZT15. Animals from the same litter were randomly assigned to the different ZT conditions. Animals were collected, and immediately sacrificed by neck dislocation and decapitation. Coronal 350 µm slices were cut using a vibrating microtome (Microm HM650V) in a pre-chilled cutting solution containing (in mM): 65 Sucrose, 85 NaCl, 2.5 KCl, 1.25 NaH_2_PO_4_, 7 MgCl_2_, 0.5 CaCl_2_, 25 NaHCO_3_ and 10 glucose, pH 7.2–7.4 and bubbled with carbogen (95% O_2_/5% CO_2_), as previously^44^. Slices were then incubated for at least 1 h in a storage chamber containing artificial cerebrospinal fluid (aCSF; in mM): 130 NaCl, 3.5 KCl, 1.2 NaH_2_PO_4_, 1 MgCl_2_, 1.5 CaCl_2_, 24 NaHCO_3_ and 10 glucose, pH 7.2–7.4, at RT and bubbled with carbogen. When required, slices were transferred to a recording chamber superfused with aCSF, bubbled with carbogen (30 °C and perfusion speed of 2 ml/min). Pharmacological manipulations were delivered by bath application of drugs through the perfusion system for at least 15 minutes. Stock solutions were generated, aliquoted, and stored at -20 °C. On an experiment day, stock solution was added to the aCSF to achieve the desired final concentration (in µM): 10 bumetanide (NKCCI inhibitor, Tocris), 10 VU0463271 (KCC2 inhibitor, Tocris).

#### Patch clamp recordings

Patch pipettes (2-5 MOhm) were pulled from standard wall borosilicate glass capillaries (Warner Instruments) with a custom-made program on a pipette puller (Sutter Instrument). Neurons were visualized under a 60x water-immersion objective (Olympus BX51WI). Recordings were performed with an Axopatch 1D amplifier (Molecular Devices), acquired using WinWCP Strathclyde software (V.3.9.7; University of Strathclyde) and stored for off-line analysis.

To measure miniature excitatory post-synaptic currents (mEPSCs), whole-cell patch clamp recordings were performed from L5 pyramidal neurons in S1 using pipettes filled with potassium-gluconate based internal solution containing (in mM): K-gluconate 126, KCl 4, Na2-ATP 4, Na-GTP 0.3, Na-phosphocreatine 10, HEPES 10. Osmolarity was adjusted to 290 mOsM and the pH to 7.35 with KOH. mEPSCs recordings were performed in voltage clamp mode from a holding potential of -70 mV, in the presence of 0.5 µM tetrodotoxin to block spiking, and 50 µM picrotoxin to block inhibitory synaptic transmission. Only recordings where the series resistance was below 40 MΩ were included and a HumBug (Quest Scientific) was used to filter out mains electrical noise at 50Hz.

To measure synaptically-evoked EPSCs and inhibitory postsynaptic currents (IPSCs), whole-cell patch clamp recordings were performed using pipettes filled with a cesium-gluconate based internal solution containing (in mM): 115 CsMeSO_3_, 4 Na_2_ATP, 0.4 Na_3_GTP, 10 Na2-phosphocreatine, 5 TEACl, 2 MgCl_2_, and 10 HEPES, 10 EGTA. Osmolarity was adjusted to 290 mOsM and pH to 7.35 with KOH. Recordings were performed in voltage clamp mode from L5 pyramidal neurons in S1, whilst an excitatory-inhibitory circuit was activated via a sharpened epoxy-insulated tungsten stimulating electrode (A-M Systems; tip <10 μm) placed in lower L2/3. EPSCs were recorded by holding the pyramidal neuron at the reversal potential for GABA_A_ receptors (E_GABAA_; -80 mV under whole-cell recording conditions). The same stimulus elicited inhibitory postsynaptic currents (IPSCs), which were revealed when the pyramidal neuron was held at the reversal potential for glutamate receptors (E_Glu_; 0 mV). Only recordings where the series resistance was below 40 MΩ were included.

To measure E_GABAA_, gramicidin perforated patch recordings^11^ were performed using pipettes filled with a high KCl based internal solution to be able to monitor the integrity of the perforated patch, containing (in mM): 135 KCl, 4 Na_2_ATP, 0.3 Na_3_GTP, 2 MgCl_2_, and 10 HEPES. Osmolarity was adjusted to 290 mOsM and pH to 7.35 with KOH. Gramicidin (Merck) was dissolved in dimethylsulfoxide (DMSO) to achieve a stock concentration of 4 mg/ml. This stock was then diluted into the internal solution on the day of the experiment to achieve a final concentration of 80 μg/ml. The resulting solution was vortexed for 40 s, sonicated for 10 s, then filtered through a 0.45 μm pore cellulose acetate membrane filter (Nalgene) and used immediately. Recordings were made when the series resistance had stabilized to approximately 100 MΩ (approximately 30 min after gigaseal formation). [Cl^-^]_i_ measurements were performed by activating GABA_A_ receptors by delivering short ‘puffs’ of GABA (Tocris, 100 μM) via a patch pipette placed in the vicinity of the cell soma and connected to a picospritzer (5–10 psi for 20–40 ms; General Valve). Puffs were delivered at a low frequency (10 s intervals) to ensure recovery of Cl^-^ homeostasis. E_GABAA_ measurements were performed in voltage clamp mode from a holding potential of -70 mV. Test voltage ramps (a saw-tooth, down-up function of 500 ms duration, with a minimum of -90 mV and maximum of -50 mV) were delivered at baseline (control ramp) and near the peak of the GABA-evoked current (GABA ramp).

To measure synaptically-evoked potentials and investigate glutamatergic long-term potentiation (LTP) under conditions that preserve transmembrane Cl^-^ gradients, gramicidin perforated patch recordings were performed as described above. Synaptic inputs were activated via a sharpened epoxy-insulated tungsten stimulating electrode (A-M Systems; tip <10 μm) placed in lower L2/3. Pulses were delivered by an isolated voltage stimulator (Digitimer, DS2), controlled via TTL pulses from the electrophysiology software. Due to the temporal filtering effects of the membrane time constant and the higher series resistance of the gramicidin perforated recordings, synaptically-evoked potentials are predicted to be slower and longer-lasting than the synaptically-evoked currents recorded in whole-cell configuration. In LTP experiments, the peak amplitude of the subthreshold monosynaptic EPSP (less than 10 mV) was monitored for a 5-10 minute baseline period at a frequency of 0.1 Hz. LTP induction consisted of 100 stimulus trains (each comprising four pre-synaptic stimuli at 100 Hz, every 10 s), using the lowest stimulus intensity required to elicit one post-synaptic spike per stimulus. Following LTP induction, responses were monitored using the same subthreshold stimulus intensity and frequency (0.1 Hz) as before LTP induction, for a period of at least 20 mins and up to 50 mins post-induction, in line with previous work^2^. LTP was measured by comparing either EPSP peak amplitude or EPSP slope from the 5 mins of baseline immediately prior to induction, to a 10-min window, 5 to 15 min following induction. EPSP slope was defined as the rise time from 20% to 80% of the EPSP peak amplitude.

### Quantification and statistical analysis

mEPSCs were analysed with Axon pCLAMP Clampfit 10.6, using a template fit method^45^. The raw traces were initially filtered by eye to remove sweeps with large fluctuations in membrane current. A low-pass filter (Bessel) at 102 Hz was applied to remove additional high frequency noise. A standard template for a mEPSC was generated by averaging 200 mEPSC events that were manually selected from a representative neuron. During event detection, the template was passed over 3 minutes of recorded data per neuron. At each position, the matching template was scaled and offset to optimise its fit with the data. Detected events were defined as crossing a template matching threshold of 4.

A neuron’s E_GABAA_ was quantified from IV curves derived from the control and GABA ramps, generated using linear fits after 100% offline series resistance correction. No difference in the series resistance was observed between the different groups (ZT3 = 93.39±11.22 MΩ, ZT15 = 88.57±11.67 MΩ, ZT3SD = 108.62±12.96 MΩ, ZT3SD+Bume = 103.32±13.18 MΩ; p=0.645, one-way ANOVA). E_GABAA_ was defined as the membrane potential at which the control and GABA ramp currents intersected, which was equivalent to the membrane potential at which the difference between the GABA and control ramp currents was equal to zero. E_GABAA_ for each data point was a mean of 5-10 E_GABAA_ measurements.

Residual depolarization was quantified as the slope of the linear fit to the post-spike depolarization. Post-spike depolarization was manually annotated.

LTP was measured by comparing either EPSP peak amplitude or slope from the 5 minutes of baseline immediately prior to induction, and a 10-minute window, 5 to 15 min after induction, unless stated otherwise. EPSP slope was measured as the rise time from 20% to 80% of the EPSP peak amplitude. Synaptic potentiation at the level of an individual neuron was assessed by comparing each EPSP peak amplitude during baseline and after induction using a Mann-Whitney test, which gave comparable results with studies that assigned potentiation using a threshold of 20% changes in synaptic response^46^.

All data analysis was performed in Matlab, while all statistical tests were performed using InStat (GraphPad). No statistical methods were used to pre-determine sample sizes, but our sample sizes are similar to those reported in previous publications^21,47–49^. Data was assessed for normality using a Kolmogorov-Smirnov test, and then the appropriate parametric or non-parametric test was performed. Appropriate correction such as the Welch’s correction were applied when comparing data with unequal standard deviations. Details of statistical tests are stated in the figure legends. All tests were two-tailed and unpaired, with a confidence level of at least a 95%. Effect size was estimated using Cohen’s D test, computed using the ‘computeCohen_d’ function in Matlab and was denoted as ‘d’. No animals were excluded from the analyses. Plotted data represent mean ± SEM. Shaded LTP plots indicate mean ± SEM.

## Key Resources Table

**Table:** 

REAGENT or RESOURCE	SOURCE	IDENTIFIER
		
Chemicals, peptides, and recombinant proteins		
GABA	Tocris	0344
Tetrodotoxin citrate	Tocris	1069
Picrotoxin	Tocris	1128
Gramicidin A	Merck	368020
Bumetanide	Tocris	3108
VU0463271	Tocris	4719
Experimental models: Organisms/strains		
C57BL/6J	Charles River	632
C57BL/6J	Envigo	057
Software and algorithms		
WinWCP	University of Strathclyde	RRID:SCR_014713
pCLAMP	Molecular Devices	RRID:SCR_011323
MATLAB	MathWorks	RRID:SCR_001622
InStat	GraphPad	RRID:SCR_000306
		

## Supplementary Material

Supplementary Material

## Data Availability

Data is available from the corresponding authors upon reasonable request. This paper does not report original code. Any additional information required to reanalyze the data reported in this paper is available from the Lead Contact upon reasonable request.
