# Supplementary material for: Sleep wake related changes in intracellular chloride regulate plasticity at glutamatergic cortical synapses
Source: Curr Biol. Author manuscript; Available in PMC 2025 Dec 1. (PMC7618421; doi:10.1016/j.cub.2025.01.050)
Supplement: Supplementary Material [file EMS211186-supplement-Supplementary_Material.pdf]

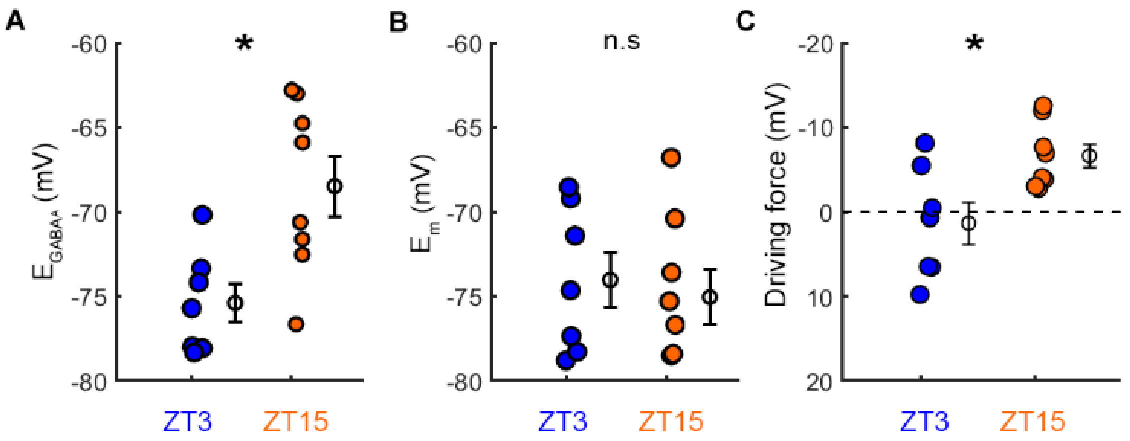

Figure S1. The active period is associated with a more depolarized E_{GABAA} and $GABA_AR$ driving force.

(A) E_{GABAA} was more depolarized at ZT15 compared to ZT3 (7 and 8 neurons, 3 and 5 animals; *p=0.014, Mann-Whitney test; d=1.62). (B) No difference was observed in the resting membrane potential (E_m; 7 and 8 neurons, 3 and 5 animals; p=0.67, t-test; t=0.44; df=13; d=0.23). (C) The $GABA_AR$ driving force, calculated as E_{GABAA} minus the neuron's resting membrane potential, was more depolarized at ZT15 than at ZT3 (7 and 8 neurons, 3 and 5 animals; *p=0.0131, t-test; t=2,87; df=13; d=1.49).

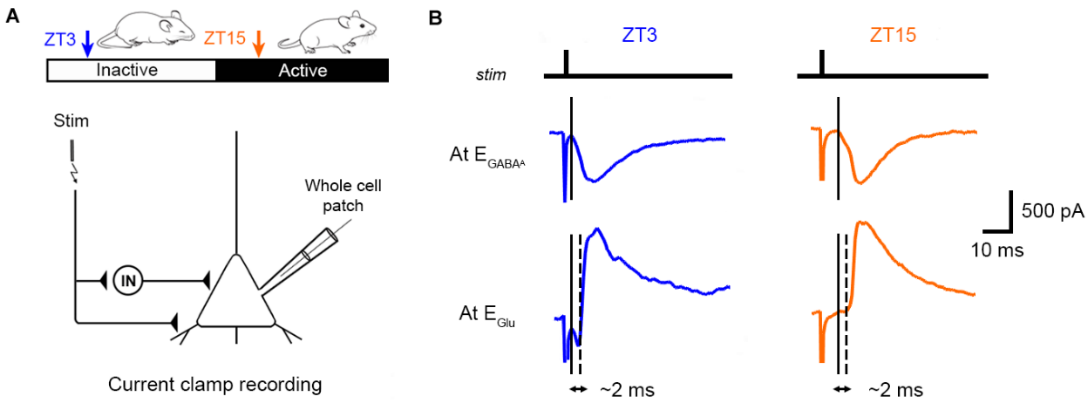

Figure S2. Stimulation in L2/3 elicits monosynaptic EPSCs and disynaptic IPSCs in L5 pyramidal neurons.

(A) Whole-cell patch clamp recordings in voltage clamp mode were performed from L5 pyramidal neurons in S1, whilst an excitatory-inhibitory circuit was activated via a stimulating electrode in lower L2/3. (B) Example recordings for a neuron at ZT3 and ZT15 show the monosynaptic EPSCs when the pyramidal neuron was held at the equilibrium potential for GABA$_A$ receptors (E_{GABAA}; -80 mV under whole-cell recording conditions). The same stimulus elicited inhibitory postsynaptic currents (IPSCs), which were revealed when the pyramidal neuron was held at the equilibrium potential for glutamate receptors (E_{Glu}; 0 mV). The timing of these IPSCs was consistent with a disynaptic delay[14].

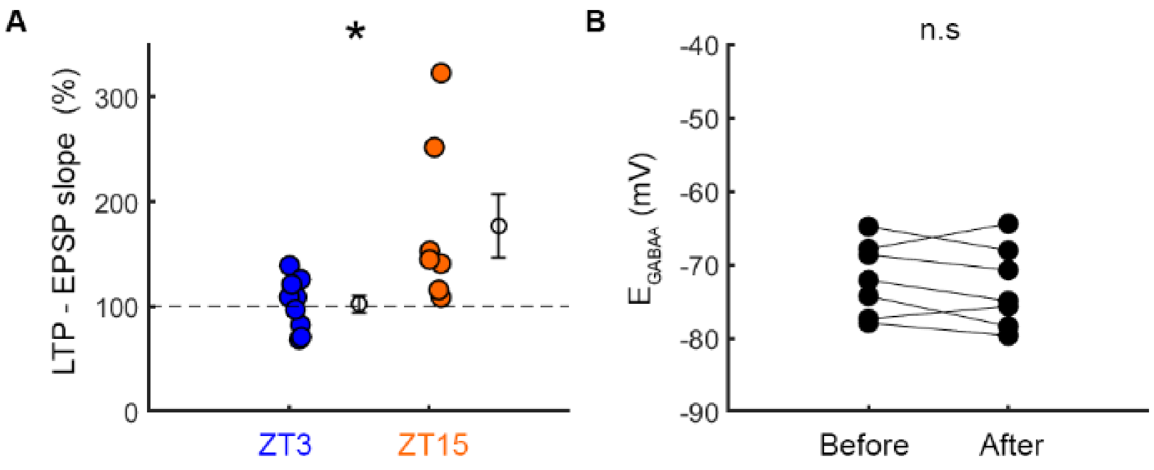

Figure S3. Depolarized E_{GABAA} during the active period is associated with glutamatergic synaptic LTP and E_{GABAA} is not affected by the LTP induction protocol.

(A) LTP was greater at ZT15 than at ZT3 when measured as the change in EPSP slope (9 and 7 neurons, 7 and 5 animals; *p=0.0115, Mann-Whitney test; d=1.34). (B) There was no significant difference in E_{GABAA} before and after LTP induction (7 neurons, 5 animals; p=0.62, Mann-Whitney test).

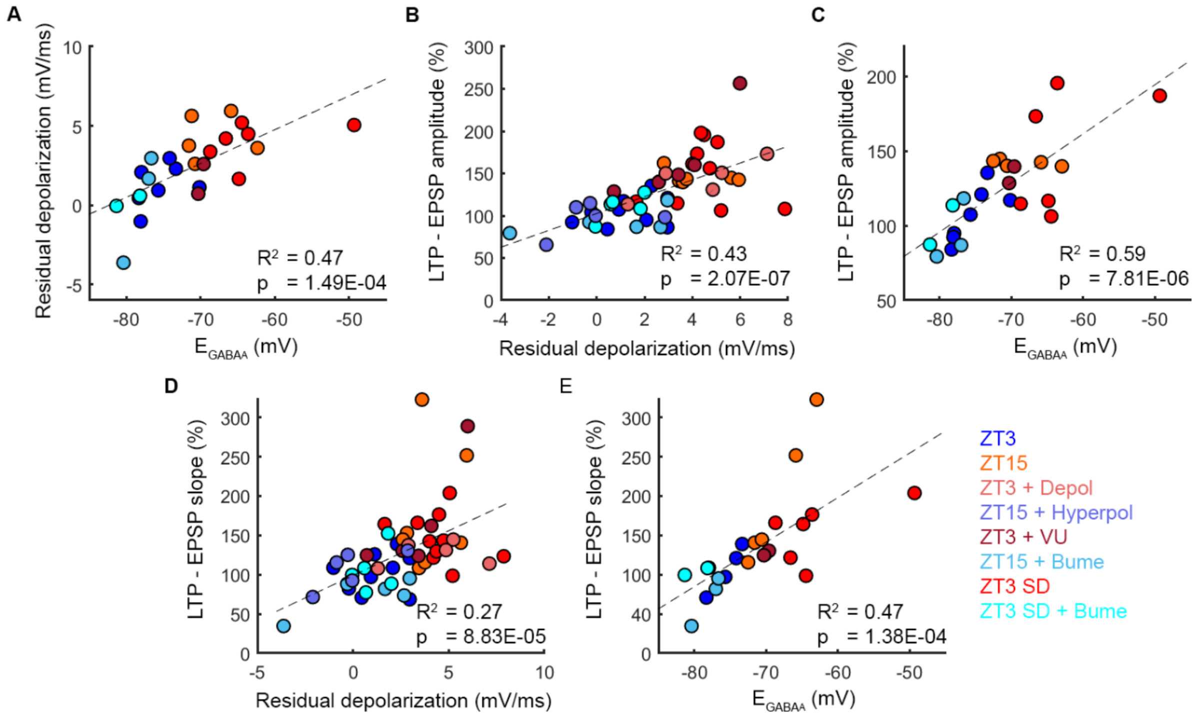

Figure S4. Across experimental conditions there are positive correlations between E_{GABAA}, residual depolarization during synaptically-evoked spiking activity, and LTP.

(A) A neuron's E_{GABAA} was correlated with the degree of residual depolarization exhibited during the synaptically-evoked spike trains (25 neurons, 21 animals; Pearson's correlation, R^2=0.47; p<0.0001). (B) A neuron's residual depolarization during LTP induction was correlated with the amount of LTP measured by EPSP peak amplitude (52 neurons, 36 animals; Pearson's correlation, R^2=0.43; p<0.0001). (C) A neuron's E_{GABAA} was correlated with the amount of LTP measured by EPSP peak amplitude (25 neurons, 21 animals; Pearson's correlation, R^2=0.59; p<0.0001). (D) A neuron's residual depolarization during LTP induction was correlated with the amount of LTP measured by EPSP slope (52 neurons, 36 animals; Pearson's correlation, R^2=0.27; p<0.0001). (E) A neuron's E_{GABAA} was correlated with the amount of LTP measured by EPSP slope (25 neurons, 21 animals; Pearson's correlation, R^2=0.47; p<0.0001).
